# Spatial distribution and management of isolated woody plants traditionally used as farmland boundary markers in Ibaraki Prefecture, Japan

**DOI:** 10.1186/2193-1801-1-57

**Published:** 2012-12-07

**Authors:** Yoshinori Tokuoka, Daisuke Hosogi

**Affiliations:** Biodiversity Division, National Institute for Agro-Environmental Sciences, 3-1-3, Kannondai, Tsukuba-shi, Ibaraki, 305-8604 Japan

**Keywords:** Field marker, Kernel density estimation, Segregation analysis, Species diversity, Rural landscape

## Abstract

Although noncrop woody plants in crop field landscapes serve multiple functions, the modernization of agriculture has threatened their preservation. In this study, isolated woody plants used as farmland boundary markers were investigated in Ibaraki Prefecture in the eastern Kanto region. A total of 2001 individuals of 50 species were found around 177 equidistantly spaced points. The most frequently used species was *Deutzia crenata* (60.7%), and the main subordinate species were *Pourthiaea villosa* (8.8%), *Euonymus japonicus* (7.7%), *Camellia sinensis* (6.8%), *Morus bombycis* (4.6%), and *Celtis sinensis* (4.2%). According to multiple kernel density estimation, all six species were estimated to have at least one core area of high presence probability. Spatial segregation analysis of those species observed more than twice indicated that the marker usage showed significant spatial heterogeneity in the region. According to managers at 32 farms, marker plants are seldom used for other purposes. Trimming frequency of markers varied among the managers, even for the same species. Most of the managers did not know the introducer, introduction year, and marker plant source, except four managers who introduced or restored the markers using *D. crenata* (*n* = 2) and *E. japonicus* (*n* = 2). These findings suggest that the regional diversity of markers reflects historic species selection. Therefore, preservation of woody plant markers must be planned based on the local characteristics of biocultural resource usage.

## Introduction

Noncrop plants traditionally have been maintained in crop field landscapes for diverse agricultural and land management purposes. For example, hedgerows have been maintained as field boundaries, fences, windbreaks, and sources for various products such as wood, firewood, and food (Baudry et al. 2000). From an ecological perspective, hedgerows help to sustain the diversity of birds (Hinsley & Bellemy 2000), small mammals (Kotzageorgis & Mason 1997), butterflies (Dover & Sparks 2000), and other invertebrates (Holland & Fahrig 2000). However, the enlargement of crop fields with the modernization of agriculture has threatened the conservation of noncrop woody plants in various agricultural landscapes (e.g., Baltensperger 1987; Burel & Baudry 1990; Petit et al. 2003). In European countries, efforts have been made to conserve noncrop vegetation (Baudry et al. 2000). To foster the sustainable use and effective preservation of noncrop plants, it is important to understand their abundance and compositional variation and the management practices used to maintain them. Although such aspects of British hedgerows have been well studied (Petit et al. 2003; Barr & Gillespie 2000; Barr et al. 2005; French & Cummins 2001; Garbutt & Sparks 2002), little research has been done on noncrop woody plant usage in other regions.

In Japan, isolated trees and hedges in various forms are also typical components of the rural landscape (Fukamachi et al. 2003; Fukamachi et al. 2011). Nakata et al. (2004) described 23 landscape patterns related to the use of isolated trees and isolated small woodlands on the western side of Lake Biwa in Shiga Prefecture. Ochiai and Takahashi (1999) reported on the preservation status of tall trees on levees along the side of paddy fields in Niigata and Shiga Prefectures. Ebisawa (1982,1996,2000) reported on the lane-side trees in lowland wet paddy fields in Shiga Prefecture. Uehara ([Bibr CR29_55][Bibr CR30_55][Bibr CR31_55]) made passing mention of the species used as isolated farmland boundary markers in specific localities. However, research on the distribution and management of noncrop woody plants in Japanese crop fields is still limited to studies conducted in central Japan. In addition, noncrop woody plants in upland field landscapes have not been investigated.

In the eastern Kanto region of Japan, isolated woody plants have been used as farmland boundary markers along traditionally maintained small upland fields. According to the Ministry of Agriculture, Forestry and Fisheries of Japan (2010), the number of agricultural management entities decreased from about 2 million in 2005 to 1.68 million in 2010, and the farmland area owned by each management entity tended to increase. This change in farmland ownership reflects the rapid consolidation of small farms, which has led to the disappearance of farmland boundaries and boundary markers. In addition, the replacement of local woody plant markers with artificial materials such as plastic and concrete pillars is another cause of woody marker disappearance. As observed in conventional hedgerows in Denmark (Aude et al. 2003), pesticide drift may have led to the loss of specific susceptible boundary marker tree species. Understanding the present regional distribution pattern and management status of isolated woody plant markers is fundamental for their preservation and monitoring in the future.

The aim of this study was to elucidate the spatial distribution patterns of isolated woody plants on farmland boundaries in Ibaraki Prefecture. Large areas of central and southern Ibaraki Prefecture are composed of flat upland fields and lowland paddy fields. Isolated woody markers are mostly observed in flat upland fields, which lack a distinct elevated grassy boundary as in paddy fields. Therefore, the distribution of farmland boundary markers was investigated in flat upland field areas. In addition, the introduction history and management status of boundary markers and other uses of the plants were investigated by interviewing land managers. Based on our findings, we suggest some means for preserving isolated woody boundary markers in the study region.

## Materials and methods

### Study site

Woody plant markers were investigated in Ibaraki Prefecture in the eastern Kanto region of Japan from August to December 2011. The study area experiences a warm temperate climate. According to the 1981–2010 statistics recorded at the Mito weather station, the mean temperature was 13.6°C and mean annual rainfall was 1353.8 mm.

**Figure 1:**
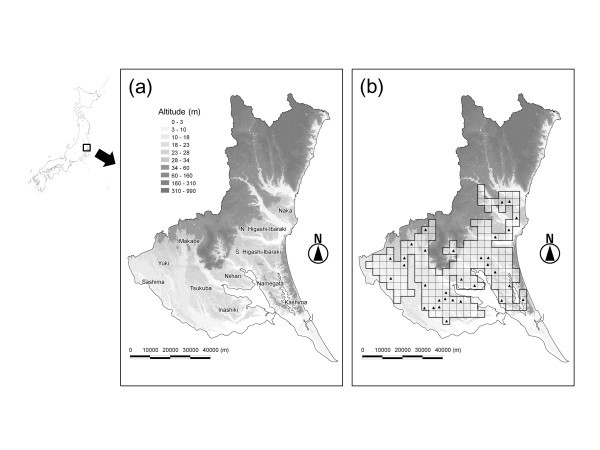
**Maps of the study site showing: (a) locations of the 10 plateaus in Ibaraki Prefecture, and (b) the 177 cells investigated for boundary marker sampling.** Triangles indicate the sites where interviews were conducted.

The central and southern parts of Ibaraki Prefecture are composed of 10 plateaus: Naka, Higashi-Ibaraki, Kashima, Namegata, Niihari, Tsukuba, Inashiki, Makabe, Yuki, and Sashima Plateaus (Figure 1a). Upland field areas are mainly distributed on the plateaus. The main crops on these plateaus are a variety of vegetables, including sweet potato, green onion, lettuce, and Chinese cabbage (Ministry of Agriculture, Forestry and Fisheries of Japan (2010)). About 70% of farmland management entities own from 0.1 to 1.0 ha of open culture vegetable fields. The upland field soil on the plateaus is mostly a well-drained volcanic Andosol with high proportions of glass and amorphous colloidal materials.

To sample farmland boundaries from at least several points on each plateau while avoiding concentrating on specific points, a systematic sampling protocol was adopted. First, 410 cells (each cell 3487 m × 3487 m) were overlaid on the prefecture area. By inspecting the land use in each cell using aerial photographs taken from 2004 to 2011 in Google Earth (http://www.google.co.jp/intl/ja/earth/index.html), cells that were mainly located within city, mountain, and lowland paddy field areas were excluded, leaving a total of 177 cells (Figure 1b).

### Boundary marker sampling

Within each cell, five farmland boundaries marked with isolated woody plants were targeted for sampling. Upland field areas close to the centroid of each cell were preliminarily checked on the aerial photographs. The area closest to the centroid was then visited, and five boundaries marked with isolated woody plants were searched. If five boundaries were not found in the first area, the area next closest to the centroid was visited, and the boundary search was continued until five boundaries were found in the cell. The farmland unit was determined at each site based on the farmland shape, which was ascertained by the plow lines, cultivated crops, and woody plant marker usage.

**Figure 2:**
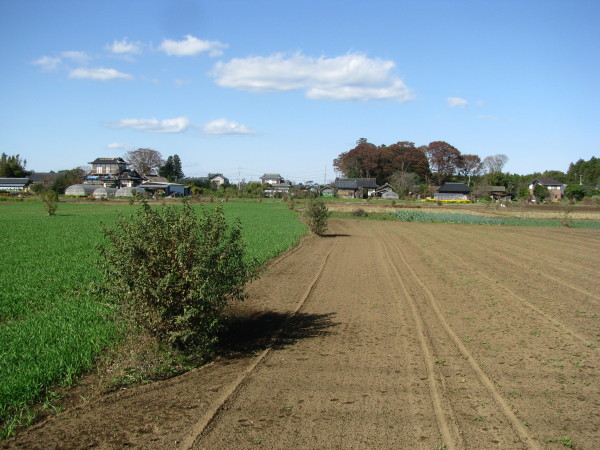
**Isolated farmland boundary markers in crop fields in Ibaraki town, central Ibaraki Prefecture.** Trimmed *Deutzia crenata* were maintained along most boundaries at the site.

An isolated woody plant was defined as a boundary marker when it was separated from the next marker plant by a gap larger than its canopy width and was clearly located on the boundary between two fields (see an example in Figure 2). In addition, when a few adjoining woody plants were rooted at one point and appeared to have been managed simultaneously, all the woody plants at that point were recorded as marker plants. Woody plants that were located on the boundary between a field and road, isolated within orchards, or part of a linear dense hedge were not included in this study because such differences in the position and structure may require particular characteristics that differ from a woody plant marker, as defined here.

The locations (longitude and latitude) of the boundary markers were recorded in World Geodetic System 84 (WGS 84) using recent photographs in Google Earth. In the statistical analysis and illustrations, longitude and latitude in WGS 84 were transformed into the coordinate system of the Japanese Geodetic Datum 2000 (JGD 2000) Zone 54 in the Universal Transverse Mercator system using Quantum GIS software version 1.7.0 (http://qgis.org/index.php). Plant nomenclature follows The Plant List (http://www.theplantlist.org/, accessed on 6 December 2011). Plant origin, distribution range, and growth form follows Miyawaki et al. (1994).

### Management status of woody plants

Semi-structured interviews were conducted at 32 farms where we met the managers during the recording of boundary markers (mostly managers of the selected boundaries, including four managers of neighboring fields; see the locations in Figure 1b). Sixteen males, 15 females, and a couple answered our questions. All managers were older than 50 years (50s, *n* = 4; 60s, *n* = 9; 70s, *n* = 14; 80s, *n* = 5). In each interview, we asked about the following predetermined items: the local name of the marker species, its introduction history (introducer, period, method, and marker plant source), its management status (method and frequency), the reason for selection of this species, and other uses. Additional information provided during the course of the conversations was also recorded.

### Statistical analysis

To inspect the spatial distribution patterns of woody markers, the presence probability of each species that was observed more than twice (28 species; Table 1) was estimated across the entire grid by multiple kernel density estimation (Diggle et al. 2005). In the estimation, a cross-validated log-likelihood function was applied to select a common bandwidth.

**Table 1:** **List of isolated woody plants used as farmland boundary markers in Ibaraki Prefecture**

Species	Family	Origin^a^	Growth form^b^	Observed number of individuals (% of total)	Number of cells in which a species was found (% of total)
*Deutzia crenata* Siebold & Zucc.	Hydrangeaceae	N	DS	1215 (60.7)	164 (92.7)
*Pourthiaea villosa* (Thunb.) Decne.	Rosaceae	N	DS	176 (8.8)	59 (33.3)
*Euonymus japonicus* Thunb.	Celastraceae	N	ES	155 (7.7)	43 (24.3)
*Camellia sinensis* (L.) Kuntze	Theaceae	A	ES	137 (6.8)	58 (32.8)
*Morus bombycis* Koidz.	Moraceae	N	DT	93 (4.6)	44 (24.9)
*Celtis sinensis* Pers.	Cannabaceae	N	DT	85 (4.2)	62 (35)
*Zelkova serrata* (Thunb.) Makino	Ulmaceae	N	DT	18 (0.9)	13 (7.3)
*Euonymus hamiltonianus* Wall.	Celastraceae	N	DS	16 (0.8)	13 (7.3)
*Celastrus orbiculatus* Thunb.	Celastraceae	N	DV	9 (0.4)	8 (4.5)
*Ligustrum lucidum* W.T. Aiton	Oleaceae	A	ET	9 (0.4)	5 (2.8)
*Ilex crenata* Thunb.	Aquifoliaceae	N	ES	7 (0.3)	5 (2.8)
*Quercus serrata* Murray	Fagaceae	N	DT	6 (0.3)	5 (2.8)
*Viburnum dilatatum* Thunb.	Adoxaceae	N	DS	6 (0.3)	5 (2.8)
*Ligustrum obtusifolium* Siebold & Zucc.	Oleaceae	N	DS	6 (0.3)	4 (2.3)
*Aphananthe aspera* (Thunb.) Planch.	Cannabaceae	N	DT	5 (0.2)	4 (2.3)
*Quercus myrsinifolia* Willd.	Fagaceae	N	ET	4 (0.2)	4 (2.3)
*Symplocos chinensis* (Lour.) Druce	Symplocaceae	N	DS	4 (0.2)	4 (2.3)
*Lindera glauca* (Siebold & Zucc.) Blume	Lauraceae	N	DS	4 (0.2)	3 (1.7)
*Eurya japonica* Thunb.	Pentaphylacaceae	N	ES/ET	4 (0.2)	2 (1.1)
*Callicarpa japonica* Thunb.	Lamiaceae	N	DS	3 (0.1)	3 (1.7)
*Abelia spathulata* Siebold & Zucc.	Caprifoliaceae	N	DS	3 (0.1)	1 (0.6)
*Euonymus alatus* (Thunb.) Siebold	Celastraceae	N	DS	2 (0.1)	2 (1.1)
*Hibiscus syriacus* L.	Malvaceae	A	DS	2 (0.1)	2 (1.1)
*Quercus acutissima* Carruth.	Fagaceae	N	DT	2 (0.1)	2 (1.1)
*Toxicodendron trichocarpum* (Miq.) Kuntze	Anacardiaceae	N	DT	2 (0.1)	2 (1.1)
*Weigela decora* (Nakai) Nakai	Caprifoliaceae	N	DS	2 (0.1)	2 (1.1)
*Wisteria floribunda* (Willd.) DC.	Leguminosae	N	DV	2 (0.1)	2 (1.1)
*Pasania edulis* (Makino) Makino	Fagaceae	N	ET	2 (0.1)	1 (0.6)
*Ailanthus altissima* (Mill.) Swingle	Simaroubaceae	A	DT	1 (0.0)	1 (0.6)
*Camellia japonica* L.	Theaceae	N	ET	1 (0.0)	1 (0.6)
*Castanea crenata* Siebold & Zucc.	Fagaceae	N	DT	1 (0.0)	1 (0.6)
*Chaenomeles sinensis* (Thouin) Koehne	Rosaceae	A	DT	1 (0.0)	1 (0.6)
*Corylopsis spicata* Siebold & Zucc.	Hamamelidaceae	NIN	DS	1 (0.0)	1 (0.6)
*Diospyros kaki* Thunb.	Ebenaceae	A	DT	1 (0.0)	1 (0.6)
*Lonicera gracilipes* Miq.	Caprifoliaceae	N	DS	1 (0.0)	1 (0.6)
*Mallotus japonicus* (L.f.) Müll. Arg.	Euphorbiaceae	N	DT	1 (0.0)	1 (0.6)
*Melia azedarach* L.	Meliaceae	NIN	DT	1 (0.0)	1 (0.6)
*Nandina domestica* Thunb.	Berberidaceae	NIN	ES	1 (0.0)	1 (0.6)
*Picrasma quassioides* (D. Don) Benn.	Simaroubaceae	N	DT	1 (0.0)	1 (0.6)
*Podocarpus macrophyllus* (Thunb.) Sweet	Podocarpaceae	N	ET	1 (0.0)	1 (0.6)
*Prunus jamasakura* Siebold ex Koidz	Rosaceae	N	DT	1 (0.0)	1 (0.6)
*Punica granatum* L.	Lythraceae	A	DT	1 (0.0)	1 (0.6)
*Pyrus pyrifolia* (Burm. f.) Nakai	Rosaceae	A	DT	1 (0.0)	1 (0.6)
*Rhus javanica* L.	Anacardiaceae	N	DT	1 (0.0)	1 (0.6)
*Rosa multiflora* Thunb.	Rosaceae	N	DS	1 (0.0)	1 (0.6)
*Salix triandra* L.	Salicaceae	N	ES/ET	1 (0.0)	1 (0.6)
*Serissa japonica* (Thunb.) Thunb.	Rubiaceae	A	ES	1 (0.0)	1 (0.6)
*Spiraea thunbergii* Siebold ex Blume	Rosaceae	NIN	DS	1 (0.0)	1 (0.6)
*Stachyurus praecox* Siebold & Zucc.	Stachyuraceae	N	DS	1 (0.0)	1 (0.6)
*Ulmus parvifolia* Jacq.	Ulmaceae	NIN	DT	1 (0.0)	1 (0.6)

^a^ N: native species, NIN: non-indigenous native species, A: alien species.

^b^ D: deciduous, E: evergreen, S: small tree, T: tall tree, V: vine.

The significance of spatial segregation of woody marker usage was tested by using a Monte Carlo test of spatial segregation in a multiple-point process (run = 1000; see details in (Diggle et al. 2005)). This analysis tested the null hypothesis of no spatial variation in the presence probability between pairs of different woody plant markers.

These analyses were conducted with the spatialkernel package developed by P. Zheng and P. Diggle (http://cran.r-project.org/web/packages/spatialkernel/index.html) using R software version 2.11.1 (R Development Core 208 Team 2010).

## Results

### Spatial distribution pattern of the markers and segregation analysis

A total of 2001 individuals of 50 woody species were recorded around the 177 grid points (Table 1). The most frequently used species was *Deutzia crenata* (60.7%), and the main subordinate species were *Pourthiaea villosa* (8.8%), *Euonymus japonicus* (7.7%), *Camellia sinensis* (6.8%), *Morus bombycis* (4.6%), and *Celtis sinensis* (4.2%). Although the well-established tea plant, *Cam*. *sinensis*, was an exception, most marker species were native plants. The growth forms of the marker species were diverse, and both deciduous and evergreen species were used. Many tall tree species and two woody vine species were used, but these were trimmed to maintain a small form.

**Figure 3:**
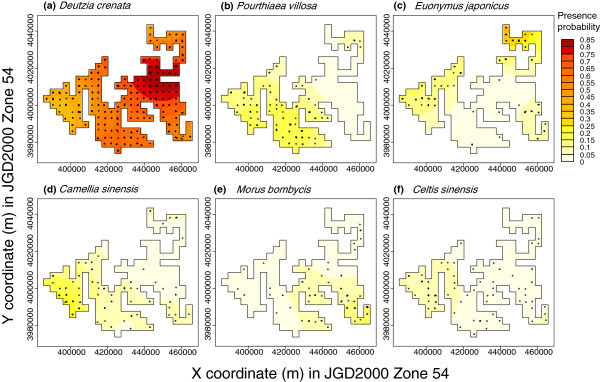
**Presence probability of the six most frequently used species.** Multiple kernel density estimation was applied to the 28 species observed more than twice. Only six species that had a more than 5% gradient in estimated presence probability are illustrated. Circles indicate the locations where each species was recorded.

The bandwidth selected for the kernel density estimation was 9800 m. *Deutzia crenata* was most frequently used on Higashi-Ibaraki Plateau (Figure 3). In the other regions, *D*. *crenata* was mostly dominant, but some of the main subordinate species were also frequently observed at specific areas. *Pourthiaea villosa* had a core distribution area on Tsukuba and Inashiki Plateaus, and that of *Cam*. *sinensis* was on Sashima Plateau. The core distribution area of *Euonymus japonicus* was on Naka Plateau. *Morus bombycis* had a core distribution area on Kashima Plateau, and that of *Cel*. *sinensis* was around Sashima and Tsukuba Plateaus. Some of the other 22 species also had a core area of high presence probability, but the estimated probability was less than 5% throughout the region.

Spatial segregation analysis of the 28 species that were observed more than twice revealed that marker species usage was significantly heterogeneous in the region (Monte Carlo test, run = 1000, *P* = 0.001).

### Management status of marker plants

The 32 interviewees managed *D*. *crenata* (*n* = 23), *P*. *villosa* (*n* = 8), *E*. *japonicus* (*n* = 3), *Cam*. *sinensis* (*n* = 5), *M*. *bombycis* (*n* = 3), *Cel*. *sinensis* (*n* = 1), *Lindera glauca* (*n* = 1), *Aphananthe aspera* (*n* = 1), and *Zelkova serrata* (*n* = 1). Some managers owned more than one species. Two woody plants were called by local names: *P*. *villosa* (standard Japanese name *Kamatsuka*) was called *Ushikoroshi* (*n* = 6), and *E*. *japonicus* (standard Japanese name *Masaki*) was called *Tamatsubaki* (*n* = 1). Two owners did not know their own marker plants’ names, and two others wrongly identified different trees as *Utsugi*, which is the standard Japanese name for *D*. *crenata*.

New marker introduction or boundary restoration was conducted by four managers; they used *D*. *crenata* obtained by dividing a neighboring marker individual (*n* = 1) and by transplanting a seedling that emerged in a neighboring forest (*n* = 1) and *E*. *japonicus* obtained by planting a cutting from a neighboring marker individual (*n* = 1) and by transplanting a seedling from an unknown source (*n* = 1). The other 28 managers did not know the introducer, introduction year, or marker plant source, and most of them have simply maintained their markers for approximately half a century (the maximum period was at least 66 years for maintenance of *D*. *crenata*). They assumed that their parents or elder ancestors introduced the markers.

Trimming of longer branches was the most common marker management. Trimming frequency varied among managers, from once every several years to several times per year. One manager has periodically cut down all aboveground parts of the marker trees.

Although the precise reasons for selecting each species were unclear because most managers did not introduce the markers themselves, some managers stated the likely reasons as local custom (*n* = 8) and strength of a species based on its ability to survive the repetitive trimming used to maintain markers at a small size (*n* = 8). Three managers stated that the woody markers can withstand damage better than plastic pillars. For example, one manager said that machine plowing severely damages and removes the head of a plastic pillar, whereas *D*. *crenata* resprouts after the aboveground part is removed by the plow.

The marker plants are seldom used for other purposes. As a local religious habit, a walking stick made of *D*. *crenata* was placed with a dead body upon interment as a part of the burial outfit of Tendai Buddhists and Shinto-style funerals at Omitama city and Ibaraki town on South Higashi-Ibaraki Plateau about 30 years ago (*n* = 2) and several years ago (*n* = 1). This ceremonial usage of the plant has not continued into recent times because funerals are usually conducted not at home but at ceremonial facilities using a wooden stick not made of *D*. *crenata*. The flowering of *D*. *crenata* was also used as an indicator of the sowing date of the vegetable *Cryptotaenia japonica* (*n* = 1), and the seeds of *D*. *crenata* were used as bullets in toy peashooters (*n* = 1). According to two managers at Tsukuba city and Ishioka city, *P*. *villosa* was frequently used along woodland boundaries. *Cam*. *sinensis* had been harvested for tea leaf production (*n* = 4). According to one manager, *Cam*. *sinensis* was maintained on a small scale around upland fields as a linear hedge or isolated tree and used for tea production in the Sashima region, which is famous for its tea production. Tea growers started cultivating *Cam*. *sinensis* on large-scale farms about 15 years ago, resulting in the decline of small-scale tea production by many local farmers.

## Discussion

Our observations indicated that *D*. *crenata* was the most frequently used woody marker among the 50 marker species in the region (Table 1). However, its presence probability was spatially heterogeneous, and the other five subordinate species (*P*. *villosa*, *E*. *japonicus*, *Cam*. *sinensis*, *M*. *bombycis*, and *Cel*. *sinensis*) were abundant in areas where *D*. *crenata* was less dominant (Figure 3). Significant spatial segregation of the 28 marker species that were observed more than twice suggested that boundary marker usage is diverse at a regional scale. Some of the pattern of segregation among the six main species seems to relate to plateau units. The clearest pattern was found for *P*. *villosa*, which is not used on Higashi-Ibaraki, Namegata, or Kashima Plateaus. In addition, the other five species each had a core distribution area on a specific plateau. According to Fujita (2002), ethno-regions of Ibaraki Prefecture were roughly divided into three groups based on the differences of dialects, the names of the same goods, and festivals and ceremonies. The northern part was segregated by the Naka River (Naka Plateau and further north), the southwestern part was segregated by the Kinu River or Kokai River (e.g., Yuki and Sashima Plateau), and the rest comprised the southeastern part. This segregation pattern is fairly consistent with the core distribution areas of some isolated woody plants, such as *Cam*. *sinensis* and *E*. *japonicus*. This suggests that topographically segregated areas share the same cultural histories, and isolated woody plant marker are representative of these cultures.

Although only the six species illustrated in Figure 3 had a distribution probability gradient of more than 5% at our sampling scale and intensity, minor marker usage may be heterogeneous at much finer local scales. Moreover, two managers in Tsukuba city and Ishioka city said that *P*. *villosa* was also frequently used along woodland boundaries. This may indicate that species selection of woodland boundary markers also has regional variation, as we found for upland field boundary markers.

Multiple uses may have contributed to the marker species selection in our study region. For example, the ritual use of *D*. *crenata* in burial ceremonies and tea leaf production from *Cam*. *sinensis* were supported by upland field boundary markers. According to Uehara (1961a), a walking stick made of *D*. *crenata* was also used in the burial ceremony in the Kita-Saku area, Nagano Prefecture, central Japan. *Cam*. *sinensis* was used as a marker most frequently in the southwestern part of the prefecture, on Sashima Plateau (Figure 3). In Ibaraki Prefecture, tea production on this plateau has been famous since the Edo era (Imai 1974). *Cam*. *sinensis* used to be planted both as linear hedges along farmland boundaries to protect wheat from wind damage and as isolated trees to mark farmland boundaries in the region (Yamazaki et al. 1977). According to one manager, such linear hedges and isolated trees had been used for small-scale tea production until about 15 years ago. Therefore, this historical local agricultural practice was reflected in the present composition of marker species.

Ebisawa (1996,2000) reported on the lane-side tree compositions at two locations in Shiga Prefecture; they found 2837 individuals representing 63 species (including six unidentified groups) on 173 paddy lanes in Nagahama city and 1001 individuals representing 59 species (including two unidentified groups) on 113 paddy lanes in Youkaichi city. Such a high diversity of lane-side trees species in the two localities would be related to the functions of the trees. The lane-side trees in Shiga Prefecture had several uses, such as *Hasagi* (drying rice after harvest using lane-side trees as pillars), harvesting fruit and materials for craftwork, and use as a landscape plant (Ebisawa 1982). To serve the various purposes, such a wide variety of trees were expected to be maintained at the local scale. In contrast, the farmland markers in upland fields in our study region have not been used directly in harvesting processes, such as *Hasagi*. As indicated by our interviews, at present woody plant markers serve only the limited role of marking farmland boundaries. This situation suggests the vulnerability of marker maintenance in the future because artificial markers are widely available as substitutes.

As stated by eight managers, species selection for farmland boundary markers is likely primarily based on the ability of the plants to withstand periodic trimming to maintain them at a small size suitable for marking upland field boundaries. According to our interviews, one *D*. *crenata* individual was maintained for at least 66 years and many other individuals of this species have survived for approximately half a century. Similarly, most individuals of the other species noted in our interviews were also maintained for approximately 50 years. These findings suggest that individuals of the main marker species that we observed can commonly persist for decades under repetitive trimming on upland field boundaries. Such persistence would be an important characteristic in marker selection. Moreover, the degree of difficulty of propagation also would be important for species selection. If vegetative propagation is easy, neighboring markers can be the source of new markers. Although most managers did not introduce markers themselves, the two managers who did so restored their markers by division of *D*. *crenata* and by planting a cutting of *E*. *japonicus*. *Euonymus japonicus* is known to be easily propagated from cuttings (Uehara 1961b). Therefore, at least these two species would be suitable for vegetative propagation in an open upland field environment.

Our study revealed the spatial segregation of boundary marker usage and the management status of these markers in Ibaraki Prefecture. The regionally diverse marker trees have an ethno-botanical value because they are characteristic features of the flat upland field landscape, which is a form of cultural heritage in the prefecture. Moreover, according to our interviews, alternative materials made of plastic or concrete are easily available. However, traditional woody markers can withstand repetitive damage during farming better than modern plastic materials. It is important that people throughout the region recognize these values of woody markers.

Marker preservation needs to be planned based on the regional variation of species usage and cultural traditions. The topographically segregated patterns of tree usage shown in Figure 3 provide beneficial information for selecting the core plateaus for marker species preservation. As noted by Fujita (2002), some ethno-regions should be recognized in advance. For example, preserving woody markers in certain areas on Naka, Higashi-Ibaraki, Kashima, Tsukuba, and Sashima Plateaus should achieve the preservation of the basic variation of marker tree usage in the region. As a similar problem has been discussed by Fukamachi et al. (2003), knowledge of marker maintenance has not been handed down well to the present managers in our study region, especially in terms of how to introduce each plant. Four managers did not know the correct name of their markers. In order to motivate the preservation of woody markers, it is important to identify other benefits they provide that would be of value to the farm managers.

To manage the remnant marker trees effectively in a context of modernization of agriculture, other possible functions for the plants should be evaluated in further research. For example, hedgerows contribute to the conservation of a variety of organisms (Barr et al. 2005). Moreover, sparse isolated trees maintained as paddock trees in Australia provide foraging sites and serve as stepping stones, thus enhancing landscape connectivity for a variety of bird species (Fischer & Lindenmayer [Bibr CR12_55][Bibr CR13_55]). Considering such ecological roles of noncrop woody plants in rural landscapes, the influence of marker selection and management on wild animal populations should be evaluated in our study region. Although our interviews revealed the importance of cultural aspects of the markers, such information for minor species remains scarce. Therefore, more intensive interviewing to learn about the marker usage in the region should be conducted in the future, which would lead to a deeper understanding of the spatial segregation and traditional cultural uses of the boundary markers.
